# Sub‐50 nm Iron–Nitrogen‐Doped Hollow Carbon Sphere‐Encapsulated Iron Carbide Nanoparticles as Efficient Oxygen Reduction Catalysts

**DOI:** 10.1002/advs.201800120

**Published:** 2018-05-12

**Authors:** Haibo Tan, Yunqi Li, Jeonghun Kim, Toshiaki Takei, Zhongli Wang, Xingtao Xu, Jie Wang, Yoshio Bando, Yong‐Mook Kang, Jing Tang, Yusuke Yamauchi

**Affiliations:** ^1^ International Center for Materials Nanoarchitectonics (WPI‐MANA) National Institute for Materials Science (NIMS) 1‐1 Namiki Tsukuba Ibaraki 305‐0044 Japan; ^2^ College of Chemistry and Molecular Engineering Qingdao University of Science and Technology Qingdao 266042 China; ^3^ Faculty of Science and Engineering Waseda University 3‐4‐1 Okubo Shinjuku Tokyo 169‐8555 Japan; ^4^ Department of Automotive Engineering School of Transportation Science and Engineering Beihang University Beijing 100191 P. R. China; ^5^ School of Chemical Engineering & Australian Institute for Bioengineering and Nanotechnology (AIBN) The University of Queensland Brisbane QLD 4072 Australia; ^6^ Australian Institute for Innovative Materials (AIIM) University of Wollongong North Wollongong NSW 2500 Australia; ^7^ Department of Energy and Materials Engineering Dongguk University‐Seoul Seoul 04620 South Korea; ^8^ Department of Plant & Environmental New Resources Kyung Hee University 1732 Deogyeong‐daero, Giheung‐gu Yongin‐si Gyeonggi‐do 446‐701 South Korea

**Keywords:** hollow carbon, iron carbide, nitrogen doping, oxygen reduction reaction, triblock copolymer templates

## Abstract

Sub‐50 nm iron–nitrogen‐doped hollow carbon sphere‐encapsulated iron carbide nanoparticles (Fe_3_C‐Fe,N/C) are synthesized by using a triblock copolymer of poly(styrene‐*b*‐2‐vinylpyridine‐*b*‐ethylene oxide) as a soft template. Their typical features, including a large surface area (879.5 m^2^ g^−1^), small hollow size (≈16 nm), and nitrogen‐doped mesoporous carbon shell, and encapsulated Fe_3_C nanoparticles generate a highly active oxygen reduction reaction (ORR) performance. Fe_3_C‐Fe,N/C hollow spheres exhibit an ORR performance comparable to that of commercially available 20 wt% Pt/C in alkaline electrolyte, with a similar half‐wave potential, an electron transfer number close to 4, and lower H_2_O_2_ yield of less than 5%. It also shows noticeable ORR catalytic activity under acidic conditions, with a high half‐wave potential of 0.714 V, which is only 59 mV lower than that of 20 wt% Pt/C. Moreover, Fe_3_C‐Fe,N/C has remarkable long‐term durability and tolerance to methanol poisoning, exceeding Pt/C regardless of the electrolyte.

## 1 Introduction

The oxygen reduction reaction (ORR) on the cathode is a major determining half‐reaction for the performance of metal–air batteries and fuel cells. Since there is a high need for Pt‐based catalysts, the search for earth‐abundant catalysts attracts tremendous interest for the purpose of extending their use and increasing their commercial efficiency.^1^, ^2^, ^3^ To date, nitrogen‐doped carbon (N/C)^4^ and iron–nitrogen‐doped carbon (Fe–N/C)^5^, ^6^, ^7^, ^8^, ^9^, ^10^ are considered highly promising candidates to replace Pt‐based catalysts toward ORR. The existence of nitrogen^11^, ^12^, ^13^, ^14^, ^15^, ^16^ and iron^6^, ^17^, ^18^, ^19^, ^20^, ^21^, ^22^, ^23^ in Fe–N/C catalysts results in enhanced ORR activity. Pyridinic N,^16^, ^24^ graphitic N,^25^ Fe–N*_x_* coordination,^26^ and iron carbide encapsulated in graphitic layers (Fe_3_C/C)^27^, ^28^, ^29^, ^30^, ^31^, ^32^ participate in the formation of active sites. Different types of nitrogen sources, Fe salts, carbon materials, and heat treatment temperatures were applied during synthesis to achieve optimal active sites for the ORR.

In addition to the chemical composition, the ORR performance is closely correlated to the morphology of the Fe–N/C catalysts. Hollow spheres have the advantages of low mass density, ample void space, and highly porous shells, thereby allowing greater active site exposure, efficient transport paths on the porous shells, and a short ion diffusion distance through the hollow center.^33^, ^34^, ^35^ Hollow Fe–N/C spheres derived from various precursors are rapidly attracting research interest and showing noteworthy ORR activities. In general, a hollow structure is obtained by using sacrificial SiO_2_ beads as a hard template that must be removed by using dangerous chemicals, such as HF and hot KOH liquid.^22^, ^36^, ^37^, ^38^, ^39^, ^40^ The hard‐templating strategy can generate hollow Fe–N/C spheres with a wide size distribution from 50 to 600 nm.^22^, ^36^, ^37^, ^38^, ^39^, ^40^ It is still difficult to realize a diameter of the spheres of less than 50 nm with an ultrasmall hollow size (less than 20 nm) because of the challenge of synthesizing small hard beads. However, applying a soft template (e.g., micelles) is a maneuverable strategy to fill the gap. Hollow Fe–N/C spheres with ultrasmall diameters can lead to increased surface areas and enhanced ORR performance.

In this study, well‐controlled Fe_3_C nanoparticles encapsulated in iron–nitrogen‐doped hollow carbon spheres with ultrasmall diameters (less than 50 nm) and hollow sizes (≈16 nm) were realized based on an asymmetric triblock copolymer of poly(styrene‐*b*‐2‐vinylpyridine‐*b*‐ethylene oxide) (PS‐*b*‐P2VP‐*b*‐PEO). Nitrogen‐rich melamine‐formaldehyde resin (M‐FR) was used as a source of carbon and nitrogen. FeCl_3_ was used as a source of Fe. The length of the PS core determines the size of the hollow center, and the concentration of the M‐FR directs the thickness of the porous shells. Although Fe–N/C catalysts show excellent ORR performance in alkaline electrolytes, few groups have reported electrocatalytic activities comparable to those of commercially available catalysts in acidic electrolytes.^26^, ^29^, ^41^ However, our sub‐50 nm hollow Fe_3_C‐Fe,N/C‐900 spheres show comparable ORR performance to that of commercially available 20 wt% Pt/C (Pt/C) in both alkaline and acidic media, which is mainly due to the following synergetic contribution of the compositions and morphologies: (i) well‐distributed Fe_3_C nanoparticles encapsulated in hollow carbon spheres; (ii) high content of pyridinic N and graphitic N that can form active sites; (iii) large surface area and porous shells that allow the exposure of active sites and rapid mass‐transfer kinetics; and (iv) small hollow size (<20 nm) and thin carbon shell (≈10 nm) that shorten the ion diffusion distance.

## 2 Results and Discussion

**Scheme 1:**
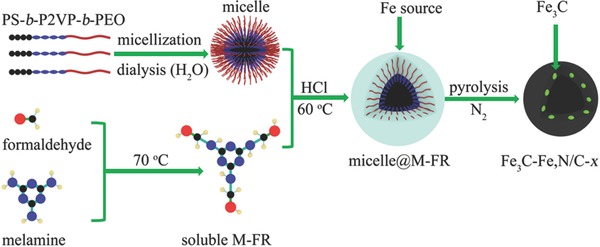
Illustration of the process necessary for preparing PS‐*b*‐P2VP‐*b*‐PEO micelle@M‐FR spheres and pyrolyzed hollow Fe_3_C‐Fe,N/C*‐x* spheres.

As shown in **Scheme** **1**, fresh soluble M‐FR was first prepared at 70 °C and mixed with the PS‐*b*‐P2VP‐*b*‐PEO micelle solution. Then, 2 m HCl works as a catalyst to stimulate the protonation of M‐FR and P2VP segment, resulting in their combination via Cl^−^.^42^, ^43^, ^44^ In this case, the combination of M‐FR and a triblock copolymer is very likely driven by the I^+^X^−^S^+^ mechanism under acidic conditions.^45^ Meanwhile, abundant hydrogen bonds between the M‐FR, P2VP, and PEO segments further facilitate their combination at 60 °C via the I^0^S^0^ (hydrogen bonding) mechanism.^46^, ^47^, ^48^, ^49^ HCl can stimulate the condensation polymerization of soluble M‐FR to generate a high‐molecular‐weight M‐FR,^50^, ^51^ so as‐prepared micelle@M‐FR composites are uniform spheres (Figures S1 and S2, Supporting Information). After the thermal decomposition of the triblock copolymer and M‐FR resin, hollow N/C spheres were generated and assigned to N/C‐*x* (*x* represents the pyrolysis temperature). To synthesize Fe_3_C nanoparticles encapsulated in hollow N/C spheres (i.e., Fe_3_C‐Fe,N/C‐*x*), the white powder of the micelle@M‐FR spheres was mixed with an FeCl_3_ solution.

**Figure 1:**
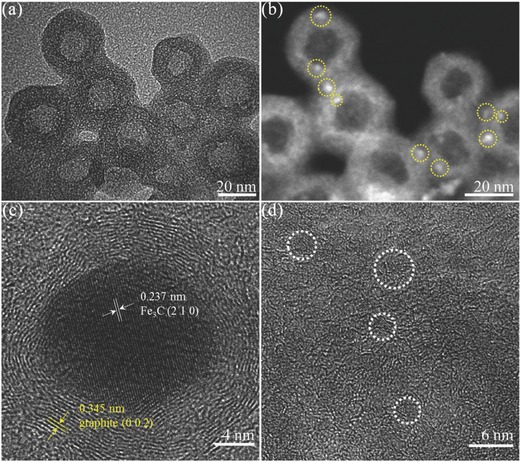
a) TEM and b) corresponding HAADF‐STEM images of Fe_3_C‐Fe,N/C‐900 hollow spheres. c) HRTEM image of a typical Fe_3_C nanoparticle surrounded by ordered graphitic layers. d) HRTEM image of Fe_3_C nanoparticles encapsulated in N‐doped carbon shells.

**Figure 2:**
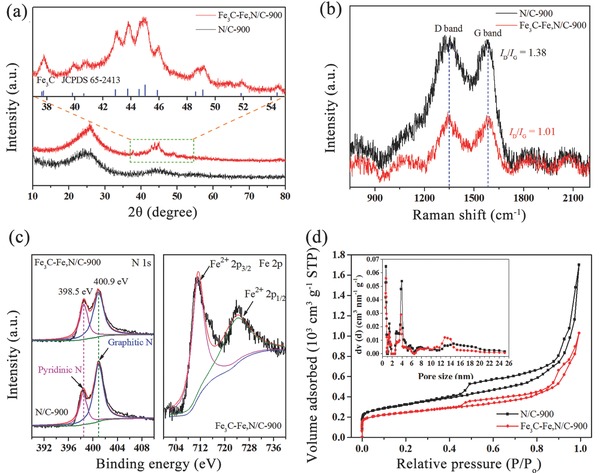
a) XRD patterns and corresponding selected spectra recorded at a scan rate of 0.1° min^−1^, b) Raman spectra, c) high‐resolution N 1s and Fe 2p XPS spectra, and d) N_2_ adsorption–desorption isotherms of Fe_3_C‐Fe,N/C‐900 and N/C‐900.

The Fe_3_C‐Fe,N/C‐900 sphere has a hollow diameter of ≈16 nm and a shell thickness of ≈10 nm (**Figure** **1**a). Its spherical morphology and hollow structure were maintained even after acid leaching and a second heat treatment at 900 °C (Figure 1; Figure S2, Supporting Information). In the high‐angle annular dark‐field scanning transmission electron microscopy (HAADF‐STEM) image (Figure 1b), the bright spots encapsulated on the N/C shell are ultrasmall Fe‐based nanoparticles (less than 4 nm). Energy‐dispersive X‐ray spectra were used to verify the presence of the Fe element. As shown in Figure S3 (Supporting Information), three typical peaks, at ≈0.70, 6.40, and 7.10 keV, can be detected on the bright spots (001 and 002 spots) but not on the gray spot (003 spot). The X‐ray diffraction (XRD) pattern shows that the Fe nanoparticles are the Fe_3_C species (JCPDS 65–2413) (**Figure** **2**a). As seen in the high‐resolution transmission electron microscopy (HRTEM) image (Figure 1c), the spacing of the central lattice fringes is ≈0.237 nm assigned to the (210) plane of the Fe_3_C phase, and the spacing of the outer lattice fringes is ≈0.345 nm assigned to the (002) plane of graphitic carbon. Crystal Fe_3_C nanoparticles are encapsulated in the graphitic carbon layers, which are well distributed in the iron–nitrogen‐doped shell (Figure 1d). The exact content of the Fe element (1.08 wt%) was determined by inductively coupled plasma‐optical emission spectrometry (ICP‐OES) analysis.

In the Raman spectra, Fe_3_C‐Fe,N/C‐900 shows a higher degree of graphitization than that of N/C‐900 (Figure 2b). The ratio of the integrated intensities of the D band to the G band (*R* = *I*_D_/*I*_G_) decreases from 1.38 to 1.01, indicating that a high content of ordered graphitic carbon was produced in Fe_3_C‐Fe,N/C‐900 due to the double heat treatment and the catalytic effect of the introduced Fe dopants.^41^, ^52^ Furthermore, a higher pyrolysis temperature can accelerate the graphitization degree, which is shown as a decreased *R* value (Figure S4b, Supporting Information). The electronic conductivity of Fe_3_C‐Fe,N/C‐900 (8771.9 S cm^−1^) measured by the four‐probe method is significantly higher than that of N/C‐900 (5.0 S cm^−1^), which should be attributed to not only the ordered graphitic carbon but also the Fe_3_C nanoparticles encapsulated into the graphitic carbon layers. Ordered graphitic carbon and Fe_3_C crystals with high electronic conductivity in Fe_3_C‐Fe,N/C‐900 can enhance the ORR activity by reducing electrochemical polarization.

The nitrogen contents of the Fe_3_C‐Fe,N/C‐900 and N/C‐900 catalysts measured by X‐ray photoelectron spectroscopy (XPS) are 6.2 and 6.9 at%, respectively. The high‐resolution N 1s spectra can be deconvoluted into two main peaks at 398.5 eV (pyridinic N) and 400.9 eV (graphitic N) (Figure 2c). Both participate in the formation of active sites.^16^, ^24^, ^25^ The high‐resolution XPS spectrum of Fe 2p can be deconvoluted into two major peaks at 710.7 eV (Fe^2+^ 2p_3/2_) and 723.6 eV (Fe^2+^ 2p_1/2_), due to the oxidation of Fe on the surface during preparation.^41^, ^53^ The signal of zero‐valent Fe, which normally has been observed in iron carbides (e.g., Fe_3_C) ≈707–708 eV, is not observed,^54^, ^55^, ^56^ further demonstrating that the Fe_3_C nanoparticles are encapsulated by graphitic carbon layers.^6^, ^57^ As shown in Figure 2d and Figure S4d (Supporting Information), the N_2_ adsorption–desorption isotherms for all samples belong to type IV with remarkable hysteresis loops, which indicates the mesoporous structure of the as‐made catalysts. As shown in Table S1 (Supporting Information), a higher pyrolysis temperature leads to an increased surface area from 805.1 to 879.5 m^2^ g^−1^ and an enlarged pore volume from 1.306 to 1.590 cm^3^ g^−1^, and more micropores were generated under higher temperatures. Hollow Fe_3_C‐Fe,N/C‐900 spheres have a high specific surface area (879.5 m^2^ g^−1^) that is lower than that of N/C‐900 (1143.0 m^2^ g^−1^) due to the contribution from metal nanoparticles. Two pore sizes are clearly observed in the mesoporous region. The smaller pore size of ≈4 nm might originate from cracks generated in the shell during pyrolysis (Figure S2b, Supporting Information), and the larger pore size of 14–20 nm corresponds to the hollow center. The high surface area, mesoporous shell, and large pore volume provide greater exposure of the active sites to electrolytes, which enhances the ORR performance.

Notably, the size of the micelle@M‐FR spheres and their assembly can be tuned by adjusting the concentration of M‐FR and the molecular weight of the PS segment. For example, 0.03 m M‐FR precursors produce micelle@M‐FR composites with an average diameter of 70 nm (Figure S5a, Supporting Information). A diameter of 90 nm was obtained when the concentration of the M‐FR precursor was increased to 0.05 m (Figure S5d, Supporting Information), while a higher concentration of the M‐FR precursor (0.10 m) resulted in aggregation of the micelle@M‐FR spheres to form super large particles (≈580 nm), which appear as mesoporous spheres (Figure S5g–i, Supporting Information). The diameter of the hollow center (Figure S5c,f, Supporting Information) is derived from the PS core. The high molecular weight of the PS segment in PS_(45 000)_‐*b*‐P2VP_(26 000)_‐*b*‐PEO_(82 000)_ enables the production of an enlarged hollow center of 85 nm in the hollow N/C‐800 spheres (Figure S6, Supporting Information). With a higher pyrolysis temperature, the size of the hollow center decreases from ≈22 nm for N/C‐800 to ≈18 nm for N/C‐900 (Figures S5f and S7, Supporting Information) due to further condensation of the structure.

The electrocatalytic activity toward the ORR of sub‐50 nm iron–nitrogen‐doped hollow Fe_3_C‐Fe,N/C‐*x* pyrolyzed at controlled temperatures was first assessed using linear sweep voltammetry (LSV). However, it should be noted that Fe_3_C‐Fe,N/C‐*x* with a much higher calcination temperatures (e.g., 950 and 1000 °C) was not obtained, since the yield under such high temperatures is only 4–5 wt% due to the self‐decomposition of the polymer precursor (Figure S8, Supporting Information). As seen in Figure S9 (Supporting Information), Fe_3_C‐Fe,N/C‐900 showed the greatest ORR activity, with the highest half‐wave potential and diffusion‐limited current density. As mentioned above, it is likely attributed to the high surface area and high graphitization degree of Fe_3_C‐Fe,N/C‐900. To better understand the catalytic activity of the Fe_3_C nanoparticles in Fe_3_C‐Fe,N/C‐900, the ORR activity of N/C‐900 was also assessed in both acidic and alkaline electrolytes. Cyclic voltammetry (CV) curves of Fe_3_C‐Fe,N/C‐900 and N/C‐900 were initially evaluated in O_2_‐ and N_2_‐saturated 0.1 m KOH and 0.1 m HClO_4_ (Figure S10, Supporting Information) at a scan rate of 50 mV s^−1^. For both samples, typical oxygen reduction peaks were observed in the O_2_‐saturated electrolyte. The well‐defined cathodic peak at 0.865 V in KOH and at 0.708 V in HClO_4_ for Fe_3_C‐Fe,N/C‐900 was superior to that for N/C‐900 (0.698 V in KOH and 0.328 V in HClO_4_). This result highlights the attribution of encapsulated Fe_3_C nanoparticles to the pronounced ORR activity.

**Figure 3:**
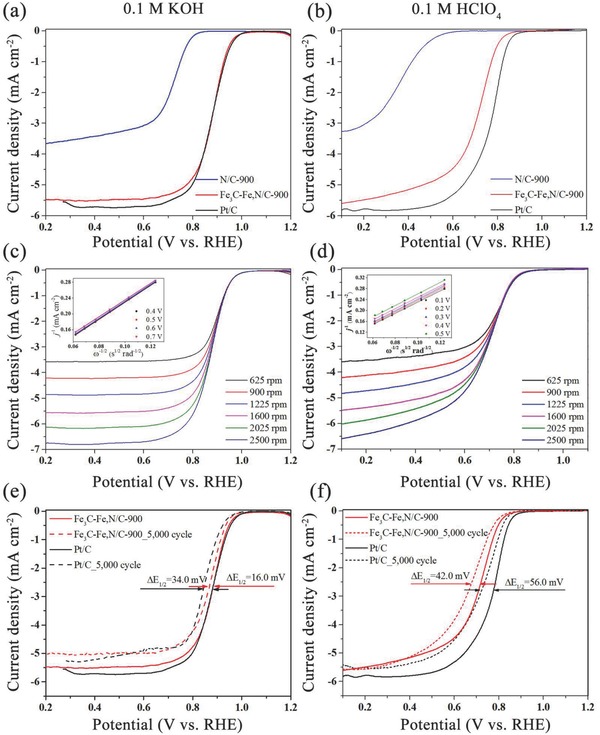
a,b) LSV curves of N/C‐900, Fe_3_C‐Fe,N/C‐900, and Pt/C at 1600 rpm and c,d) LSV curves of Fe_3_C‐Fe,N/C‐900 at different rotating speeds in 0.1 m KOH and 0.1 m HClO_4_ (insets are the corresponding Koutecky–Levich plots), respectively. e,f) LSV curves of Fe_3_C‐Fe,N/C‐900 and Pt/C before and after 5000 cycles in 0.1 m KOH and 0.1 m HClO_4_ at 1600 rpm, respectively. The scan rate is 10 mV s^−1^.

The electrocatalytic activity of three catalysts (N/C‐900, Fe_3_C‐Fe,N/C‐900, and Pt/C) toward the ORR was assessed by using a rotating disk electrode and a rotating ring‐disk electrode (RRDE) in both alkaline and acidic media. To compare their realistic performance, the loading amounts on the electrode were the same at 0.2 mg cm^−2^. In 0.1 m KOH, the LSV curve of Fe_3_C‐Fe,N/C‐900 almost overlapped with the curve of commercially available Pt/C (**Figure** **3**a). Fe_3_C‐Fe,N/C‐900 exhibited a high half‐wave potential of ≈0.881 V, which is comparable to that of commercially available Pt/C (*E*_1/2_ = 0.878 V). Poor ORR performance was observed on N/C‐900 with a negative shift in the half‐wave potential to ≈0.719 V. Compared to previously reported remarkable nitrogen‐doped carbon‐encapsulated Fe_3_C catalysts (Table S2, Supporting Information), Fe_3_C‐Fe,N/C‐900 still exhibited noticeable ORR electrocatalytic activity in alkaline media.^28^, ^29^, ^32^, ^58^ However, in 0.1 m HClO_4_, Fe_3_C‐Fe,N/C‐900 exhibited a slightly negative shift in the LSV curve. The half‐wave potential of Fe_3_C‐Fe,N/C‐900 was ≈0.714 V, which approaches the value of Pt/C of ≈0.773 V (Figure 3b). In addition, Fe_3_C‐Fe,N/C‐900 exhibited a diffusion‐limited current density of 5.35 mA cm^−2^ at 0.3 V, which is close to 5.83 mA cm^−2^ in the Pt/C catalyst. Such good ORR activity of iron–nitrogen‐doped carbon‐encapsulated Fe_3_C catalysts in an acidic medium has rarely been reported.^29^, ^30^, ^31^, ^32^, ^59^, ^60^, ^61^, ^62^

To investigate the ORR kinetics in greater detail, LSV curves of Fe_3_C‐Fe,N/C‐900 under various rotating speeds from 625 to 2500 rpm were detected in O_2_‐saturated 0.1 m KOH and 0.1 m HClO_4_ (Figure 3c,d). The corresponding Koutecky–Levich plots show fairly good linearity. The number of transferred electrons (*n*) was calculated to be 3.98–4.05 between 0.4 and 0.7 V in 0.1 m KOH. This is an ideal four‐electron pathway for the reduction of O_2_ to OH^−^ (Figure 3c). From RRDE experiments, the *n* values for Fe_3_C‐Fe,N/C‐900, Pt/C, and N/C‐900 were calculated based on Equation (3). Fe_3_C‐Fe,N/C‐900 had an *n* value of 3.91–3.98 in the potential range of 0.2–0.8 V, which is approaching the *n* value of 3.92–3.96 for Pt/C but is higher than that for N/C‐900 of 2.30–3.46 (Figure S11a, Supporting Information). Furthermore, the H_2_O_2_ yield was also monitored (Figure S11c,d, Supporting Information). Both Fe_3_C‐Fe,N/C‐900 and Pt/C showed low H_2_O_2_ yields of less than 5%; however, the H_2_O_2_ yield for N/C‐900 was over 27%. This suggests that the presence of Fe_3_C nanoparticles tremendously promotes the four‐electron ORR pathway in alkaline media. In acidic media, there is a fully efficient four‐electron pathway on Fe_3_C‐Fe,N/C‐900 with an *n* value of 4.04–4.13 between 0.2 and 0.5 V (Figure 3d). Based on the RRDE experiment, the four‐electron reaction on Fe_3_C‐Fe,N/C‐900 (*n =* 3.97) and Pt/C (*n =* 3.98) was confirmed (Figure S11b, Supporting Information), while the reaction on N/C‐900 followed a mixture reaction with two and four electrons with an *n* value of 2.33–2.74 between 0.1 and 0.55 V. These results reveal a good ORR performance from Fe_3_C‐Fe,N/C‐900 under alkaline and acidic conditions.

Much attention has been paid recently to the features of long‐term stability and methanol poisoning in fuel‐cell technology. The long‐term stability of the Fe_3_C‐Fe,N/C‐900 and Pt/C catalysts was assessed following an accelerated durability test protocol based by cycling the potential between 0.6 and 1.0 V in O_2_‐saturated 0.1 m KOH and between 0.5 and 0.9 V in O_2_‐saturated 0.1 m HClO_4_, respectively. LSV curves at a rotation speed of 1600 rpm were compared before and after 5000 continuous cycles. In 0.1 m KOH, Fe_3_C‐Fe,N/C‐900 exhibited better durability (Figure 3e). The half‐wave potential of Fe_3_C‐Fe,N/C‐900 slightly decreased by only ≈16.0 mV, whereas commercially available Pt/C underwent a half‐wave potential decrease of ≈34.0 mV. Similarly, the superiority of Fe_3_C‐Fe,N/C‐900 was observed in 0.1 m HClO_4_ (Figure 3f). The decreased half‐wave potential of ≈42 mV on Fe_3_C‐Fe,N/C‐900 shows improved stability of the ORR performance when compared to Pt/C (a half‐wave potential decrease of ≈56 mV). Furthermore, the XRD patterns for Fe_3_C‐Fe,N/C‐900 through 5000 continuous cycles still showed clear signals of Fe_3_C crystals (Figure S12, Supporting Information), demonstrating the outstanding stability of the Fe_3_C crystals surrounded by graphitic carbon layers. These results prove that homogeneously dispersed Fe_3_C protected by graphitic carbon layers can greatly improve the ORR performance and long‐term stability, regardless of the electrolyte used. The tolerance to methanol poisoning of Fe_3_C‐Fe,N/C‐900 and Pt/C catalysts was estimated in O_2_‐saturated 0.1 m KOH. After the addition of 3 m methanol, the current in the Pt/C catalyst changed from negative to positive due to the occurrence of a major methanol oxidation reaction (Figure S13, Supporting Information). By contrast, the Fe_3_C‐Fe,N/C‐900 catalyst still maintained a high activity toward the ORR, as evidenced by the nonobvious change in the ORR current, which exhibited an excellent tolerance to methanol poisoning in alkaline conditions, highlighting its potential use in fuel‐cell technology.

According to the above‐described results, Fe_3_C‐Fe,N/C‐900 exhibited superior ORR electrocatalytic activity for ORR in both alkaline and acidic media. On one hand, we speculate that such enhanced ORR activity of Fe_3_C‐Fe,N/C‐900 can be attributed to the synergistic effect between enriched pyridinic N‐doped and graphitic N‐doped carbon and Fe_3_C nanoparticles. It was reported by Bao and co‐workers that iron carbides (Fe*_x_*C*_y_*) can tune the redox properties of the surrounding carbon layer via the confinement effect.^63^ Their density function theory (DFT) calculations revealed charge transfer from Fe to the C nanotube. This type of interaction between the shielded Fe and the C allowed the absorption of O_2_ and significantly enhanced the ORR activity of the CNTs.^64^ Remarkably, the first‐principles calculations carried out by Pan and co‐workers showed that electrons are transferred from Fe_3_C to nitrogen‐doped carbon layers, resulting in a positive effect on the catalysis of nitrogen‐doped carbon layers.^65^ Meanwhile, their DFT calculations showed that a pristine and nitrogen‐doped graphene surface can spontaneously adsorb O_2_ molecules, facilitating diffusion to the active sites. Additionally, the CV and LSV curves show the degradation of ORR activity due to breaking the graphitic layers and etching Fe_3_C phases (Figure S14, Supporting Information), convincingly verifying the synergetic effect of Fe_3_C/C on ORR.^29^, ^66^ As mentioned before, pyridinic and graphitic N also participate in the formation of active sites toward the ORR.^16^, ^24^, ^25^ DFT calculations also demonstrated that doping N atoms into the carbon lattice in the region below where iron is can reduce the free energy of O_2_ sorption.^64^ Therefore, in the present study, pyridinic and graphitic N‐enriched carbon shells with encapsulated Fe_3_C nanoparticles should effectively promote ORR activity. However, Fe loading in Fe_3_C‐Fe,N/C‐900 (1.08 wt%) is lower than in a previously reported Fe_3_C‐based catalyst but with enhanced ORR performance,^28^, ^29^, ^30^, ^57^, ^67^, ^68^ which indirectly proves the enhancement from sub‐50 nm N‐doped carbon spheres with hollow and porous structures. A high surface area allows more exposure of the active sites. Meanwhile, porous thin carbon shells and sub‐20 nm (≈16 nm) hollow sizes facilitate the mass‐transfer of ORR‐related species, improving ORR performance regardless of the electrolytes used.

## 3 Conclusion

In summary, we have developed a versatile and facile route for synthesizing sub‐50 nm, hollow, iron–nitrogen‐doped carbon sphere‐encapsulated Fe_3_C nanoparticles (Fe_3_C‐Fe,N/C‐900). The size of the hollow center and the shell thickness can be controlled readily by the length of the PS core and the concentration of the M‐FR precursor, respectively. Nitrogen‐rich content and Fe_3_C nanoparticles combined with the typical structural features of the hollow carbon spheres, including a large surface area (879.5 m^2^ g^−1^), porous shells, and small hollow sizes (≈16 nm), produce easily accessible active sites, rapid mass‐transfer, and active ORR performance. Thus, Fe_3_C‐Fe,N/C‐900 hollow spheres show ORR activity comparable to that of commercially available Pt/C in both alkaline and acidic media, with similar half‐wave potential, an electron transfer number close to 4, and a low H_2_O_2_ yield of less than 5%. Moreover, Fe_3_C‐Fe,N/C‐900 has remarkable long‐term durability and tolerance to methanol poisoning, which are superior to those of commercially available Pt/C, regardless of the electrolyte.

## 4 Experimental section

*Materials*: Triblock copolymer PS_(20 000)_‐*b*‐P2VP_(15 000)_‐*b*‐PEO_(27 000)_ and PS_(45 000)_‐*b*‐P2VP_(26 000)_‐*b*‐PEO_(82 000)_ were purchased from Polymer Source. Tetrahydrofuran (THF), 2 m hydrochloric acid, a formaldehyde solution (37%), and iron(III) chloride hexahydrate (FeCl_3_·6H_2_O) were purchased from Nacalai Tesque. Melamine was purchased from Sigma‐Aldrich. All chemicals were used directly without further purification.

*Synthesis of PS‐b‐P2VP‐b‐PEO Micelle Aqueous Solution*: Typically, 0.125 g of triblock copolymer PS‐*b*‐P2VP‐*b*‐PEO was completely dissolved in 24.5 mL of THF. Then, 500 µL of a 35% HCl solution was added to stimulate micellization under stirring. A milk‐white suspension was obtained and transferred into a dialysis membrane tube (*M*_w_ cut‐off: 14 000 Da) and dialyzed against distilled water for several cycles to remove the THF and HCl. After the dialysis process, a clear PS‐*b*‐P2VP‐*b*‐PEO micelle aqueous solution was obtained, and the concentration was ≈3.6 mg mL^−1^.

*Synthesis of Hollow N/C and Fe_3_C‐Fe,N/C*: The soluble melamine resin solution was prepared first. A low‐molecular‐weight melamine resin was generated by a typical process. Then, 0.252 g of melamine was mixed with 640 µL of a formaldehyde (37 wt%) aqueous solution and 2 mL of H_2_O and reacted at 70 °C under stirring for 5 min. Afterward, 40 mL of H_2_O was added to the above clear solution. After cooling, 4.7 mL of the PS‐*b*‐P2VP‐*b*‐PEO micelle aqueous solution was added to the above solution, and the initial pH was adjusted to 4.70 ± 0.05 by adding a 2 m HCl aqueous solution. The mixed solution was then reacted at 60 °C under stirring for 12 h. The generated white precipitate was collected by centrifugation (25 000 rpm for 10 min) and washed twice with water and ethanol. After drying at room temperature, the white powder was pyrolyzed at a controlled temperature (700, 800, and 900 °C) in a N_2_ atmosphere. The ramping rate of the calcination process was 2 °C min^−1^, and it was maintained at 400 °C and a target temperature for 0.5 and 2 h, respectively. The carbonized product was named N/C‐*x*, where *x* represents the calcination temperature (700, 800, and 900 °C).

To introduce the Fe dopant, the solvent‐impregnation method was adopted to mix the white powder described above and a certain amount of a 100 × 10^−3^ m FeCl_3_ solution. After drying, the mixture was calcined at a controlled temperature (700, 800, and 900 °C). The carbonized product was leached in 0.5 m H_2_SO_4_ at 80 °C for 8 h under stirring to remove inactive metal iron particles; a second heat treatment was operated in a N_2_ atmosphere at the same temperatures with a ramping rate of 5 °C min^−1^. The final carbonized product was designated Fe_3_C‐Fe,N/C‐*x*.

*Characterization*: Field emission scanning electron microscopy (HITACHI SU‐8000) with an accelerating voltage of 5 kV was used to observe the morphology of the as‐prepared materials. A transmission electron microscope (TEM, JEOL JEM‐2100F) operated at 200 kV was employed to investigate the hollow structure and obtain detailed information about the Fe‐based nanoparticles. The exact Fe content of the pyrolyzed Fe_3_C‐Fe,N/C material was measured by ICP‐OES (SPS3520UV‐DD, Hitachi High‐Technologies Co.). Wide‐angle XRD patterns were acquired with a Rigaku Rint‐2000 X‐ray diffractometer using monochromated Cu Kα radiation (40 kV, 40 mA) at a scanning rate of 1° min^−1^. Raman spectra were recorded on a micro Raman spectrophotometer (Horiba Jobin Yvon T64000). The nitrogen adsorption–desorption isotherms were measured using a BELSORP‐max (BEL, Japan) at 77 K. The surface areas (S) and pore volumes (V) (including the micropore volume, *V*_micro_) were obtained with the Brunauer–Emmett–Teller method, the *t*‐plot method, and the nonlocal density functional theory method. The chemical states of nitrogen and iron were investigated using X‐ray photoelectron spectroscopy (PHI Quantera SXM) with Al Kα radiation (20 kV, 5 mA). The shift in the binding energy was calibrated using the C 1s level at 284.5 eV. The thermal stability of the polymer precursor was assessed by thermogravimetric analysis (6300 TG/DTA, Hitachi HT‐Seiko Instrument Exeter) under a N_2_ atmosphere.

*Electrochemical Measurements*: All as‐prepared catalysts were ground before being used as electrocatalyst inks. Commercial Pt/C (20 wt%, from Alfa Aesar, USA) was used for comparison. Typically, 5 mg of catalyst was dispersed in 1 mL of 1:2 (v/v) isopropanol/water mixed solvent (containing 0.05 mL of a 5.0 wt% Nafion solution) under sonication for at least 30 min to form a homogenous catalyst ink. Then, 5 µL of the electrocatalyst ink was dropped on the surface of a glassy carbon (GC) electrode 4 mm in diameter (RRDE Pt Ring/GC Disk Electrode, ALS Co., Ltd.) prepolished with 1 µm and 0.05 µm polishing alumina and dried at room temperature. The loading amounts of the as‐prepared catalysts and commercial Pt/C catalyst were 0.2 mg cm^−2^ for both alkaline and acidic media.

*Electrochemical Analyses*: Electrochemical analyses were performed with a CHI 842B electrochemical analyzer (CH Instruments, USA). A conventional three‐electrode cell was used, including a saturated calomel electrode (SCE) as the reference electrode, a Pt wire as the counter electrode, and the catalyst film‐coated RRDE as the working electrode. Electrochemical experiments were conducted in O_2_/N_2_ saturated 0.1 m KOH or 0.1 m HClO_4_ electrolytes, and the flow of O_2_/N_2_ was maintained over the electrolytes during the test. All potential values used in this study were normalized to the reversible hydrogen electrode (RHE), which was calculated according to the Nernst equation (*E*_RHE_ = *E*(SCE) + 0.0591* pH + 0.241). All CV measurements were conducted at a scan rate of 50 mV s^−1^ from 1.2 to 0.0 V. RRDE measurements were conducted by LSV in the same potential range at rotating speeds ranging from 625 to 2500 rpm. The disk electrode was scanned at a rate of 10 mV s^−1^, and the ring electrode potential was held at 1.2 V. Durability tests of the Fe_3_C‐Fe,N/C‐900 and Pt/C catalysts were carried out using the accelerated durability test protocol by cycling the catalysts between 0.6 and 1.0 V in O_2_‐saturated 0.1 m KOH and between 0.5 and 0.9 V in O_2_‐saturated 0.1 m HClO_4_ at a scan rate of 50 mV s^−1^, respectively. All current densities in this work were calculated after correction of the double‐layer capacitance based on the geometric area (0.1256 cm^2^) of the rotating disk electrode.

The electron transfer numbers were calculated using the Koutecky–Levich equation

(1) $$\frac{1}{j}=\frac{1}{j_{L}}+\frac{1}{j_{K}}=\frac{1}{B\omega^{1/2}}+\frac{1}{j_{K}}$$

(2) $$B=0.62nFC_{0}D_{0}^{2/3}\upsilon^{-1/6}$$

where ω is the angular velocity, *j* is the measured current density, *j*_K_ and *j*_L_ are the kinetic and diffusion‐limited current densities, respectively, *n* is the number of the transferred electrons, *F* is the Faraday constant (96 485 C mol^−1^), *C*_0_ is the bulk concentration of O_2_ (1.2 × 10^−6^ mol cm^−3^), *D*_0_ is the diffusion coefficient of O_2_ (1.9 × 10^−5^ cm^2^ s^−1^), and υ is the kinematic viscosity of the electrolyte (0.01 cm^2^ s^−1^). *B* can be derived from the slope of the K–L equation.

The following equations were used to calculate the number of transferred electron (*n*) and the hydrogen peroxide yield (%H_2_O_2_, the percentage of H_2_O_2_) released during ORR, respectively

(3) $$n=\frac{4I_{d}}{I_{d}+(I_{r}/N)}$$

(4) $$\%H_{2}O_{2}=200\;\;\times \;\;\frac{I_{r}/N}{I_{d}+\left(I_{r}/N\right)}$$

where *I*_d_ is the Faradaic current at the disk, and *I*_r_ is the Faradaic current at the ring; the H_2_O_2_ collection coefficient *N* of the ring is 0.4.

*Tolerance to Methanol*: The tolerance to methanol of the as‐made Fe_3_C‐Fe,N/C‐900 catalyst and the Pt/C was estimated by the amperometric current–time chrono‐amperometric response measurement in O_2_‐saturated 0.1 m KOH + 3 m CH_3_OH. First, the *i–t* chrono‐amperometric curve was obtained in an O_2_‐saturated 0.1 m KOH solution for 600 s at 0.65 V versus RHE at 1600 rpm, followed by the sequential addition of 3 m methanol. Then, the *i–t* chrono‐amperometric curve was obtained for another 600 s.

## Conflict of Interest

The authors declare no conflict of interest.

## Supporting information

Supplementary

## References

[1] G. Wu , K. L. More , C. M. Johnston , P. Zelenay , Science 2011, 332, 443. 21512028 10.1126/science.1200832

[2] A. Zitolo , V. Goellner , V. Armel , M. T. Sougrati , T. Mineva , L. Stievano , E. Fonda , F. Jaouen , Nat. Mater. 2015, 14, 937. 26259106 10.1038/nmat4367

[3] L. Shang , H. Yu , X. Huang , T. Bian , R. Shi , Y. Zhao , G. I. N. Waterhouse , L.‐Z. Wu , C.‐H. Tung , T. Zhang , Adv. Mater. 2016, 28, 1668. 26677131 10.1002/adma.201505045

[4] K. Gong , F. Du , Z. Xia , M. Durstock , L. Dai , Science 2009, 323, 760. 19197058 10.1126/science.1168049

[5] J. Liu , E. Li , M. Ruan , P. Song , W. Xu , Catalysts 2015, 5, 1167.

[6] W.‐J. Jiang , L. Gu , L. Li , Y. Zhang , X. Zhang , L.‐J. Zhang , J.‐Q. Wang , J.‐S. Hu , Z. Wei , L.‐J. Wan , J. Am. Chem. Soc. 2016, 138, 3570. 26906342 10.1021/jacs.6b00757

[7] Y. Chen , S. Ji , Y. Wang , J. Dong , W. Chen , Z. Li , R. Shen , L. Zheng , Z. Zhuang , D. Wang , Y. Li , Angew. Chem. 2017, 129, 7041; 10.1002/anie.201702473 28402604

[8] Q. Wang , L. Shang , R. Shi , X. Zhang , Y. Zhao , G. I. N. Waterhouse , L.‐Z. Wu , C.‐H. Tung , T. Zhang , Adv. Energy Mater. 2017, 7, 1700467.

[9] Q. Wang , L. Shang , R. Shi , X. Zhang , G. I. N. Waterhouse , L.‐Z. Wu , C.‐H. Tung , T. Zhang , Nano Energy 2017, 40, 382.

[10] P. Song , H. M. Barkholtz , Y. Wang , W. Xu , D. Liu , L. Zhuang , Sci. Bull. 2017, 62, 1602. 10.1016/j.scib.2017.10.020 36659478

[11] L. Qu , Y. Liu , J.‐B. Baek , L. Dai , ACS Nano 2010, 4, 1321. 20155972 10.1021/nn901850u

[12] D. Geng , Y. Chen , Y. Chen , Y. Li , R. Li , X. Sun , S. Ye , S. Knights , Energy Environ. Sci. 2011, 4, 760.

[13] J. Liang , X. Du , C. Gibson , X. W. Du , S. Z. Qiao , Adv. Mater. 2013, 25, 6226. 23963824 10.1002/adma.201302569

[14] W. He , C. Jiang , J. Wang , L. Lu , Angew. Chem. 2014, 126, 9657;

[15] W. Wei , H. Liang , K. Parvez , X. Zhuang , X. Feng , K. Müllen , Angew. Chem. 2014, 126, 1596; 10.1002/anie.201307319 24459087

[16] D. Guo , R. Shibuya , C. Akiba , S. Saji , T. Kondo , J. Nakamura , Science 2016, 351, 361. 26798009 10.1126/science.aad0832

[17] M. Lefèvre , E. Proietti , F. Jaouen , J.‐P. Dodelet , Science 2009, 324, 71. 19342583 10.1126/science.1170051

[18] M. S. Thorum , J. M. Hankett , A. A. Gewirth , J. Phys. Chem. Lett. 2011, 2, 295.

[19] Y. Li , W. Zhou , H. Wang , L. Xie , Y. Liang , F. Wei , J.‐C. Idrobo , S. J. Pennycook , H. Dai , Nat. Nanotechnol. 2012, 7, 394. 22635099 10.1038/nnano.2012.72

[20] D. Singh , K. Mamtani , C. R. Bruening , J. T. Miller , U. S. Ozkan , ACS Catal. 2014, 4, 3454.

[21] W. Li , J. Wu , D. C. Higgins , J.‐Y. Choi , Z. Chen , ACS Catal. 2012, 2, 2761.

[22] G. A. Ferrero , K. Preuss , A. Marinovic , A. B. Jorge , N. Mansor , D. J. L. Brett , A. B. Fuertes , M. Sevilla , M.‐M. Titirici , ACS Nano 2016, 10, 5922. 27214056 10.1021/acsnano.6b01247

[23] J. Liu , X. Sun , P. Song , Y. Zhang , W. Xing , W. Xu , Adv. Mater. 2013, 25, 6879. 24105708 10.1002/adma.201302786

[24] S. Yang , X. Feng , X. Wang , K. Müllen , Angew. Chem. 2011, 123, 5451; 10.1002/anie.201100170 21557411

[25] R. Liu , D. Wu , X. Feng , K. Müllen , Angew. Chem. 2010, 122, 2619; 10.1002/anie.200907289 20217877

[26] Y. J. Sa , D. J. Seo , J. Woo , J. T. Lim , J. Y. Cheon , S. Y. Yang , J. M. Lee , D. Kang , T. J. Shin , H. S. Shin , H. Y. Jeong , C. S. Kim , M. G. Kim , T. Y. Kim , S. H. Joo , J. Am. Chem. Soc. 2016, 138, 15046. 27750429 10.1021/jacs.6b09470

[27] Z. Wen , S. Ci , F. Zhang , X. Feng , S. Cui , S. Mao , S. Luo , Z. He , J. Chen , Adv. Mater. 2012, 24, 1399. 22311518 10.1002/adma.201104392

[28] J.‐S. Lee , G. S. Park , S. T. Kim , M. Liu , J. Cho , Angew. Chem. 2013, 125, 1060; 10.1002/anie.201207193 23315903

[29] Y. Hu , J. O. Jensen , W. Zhang , L. N. Cleemann , W. Xing , N. J. Bjerrum , Q. Li , Angew. Chem. 2014, 126, 3749; 10.1002/anie.201400358 24554421

[30] W. Yang , X. Liu , X. Yue , J. Jia , S. Guo , J. Am. Chem. Soc. 2015, 137, 1436. 25607754 10.1021/ja5129132

[31] M. Xiao , J. Zhu , L. Feng , C. Liu , W. Xing , Adv. Mater. 2015, 27, 2521. 25757871 10.1002/adma.201500262

[32] K. Strickland , E. Miner , Q. Jia , U. Tylus , N. Ramaswamy , W. Liang , M.‐T. Sougrati , F. Jaouen , S. Mukerjee , Nat. Commun. 2015, 6, 7343. 26059552 10.1038/ncomms8343 PMC4490352

[33] F. Xu , Z. Tang , S. Huang , L. Chen , Y. Liang , W. Mai , H. Zhong , R. Fu , D. Wu , Nat. Commun. 2015, 6, 7221. 26072734 10.1038/ncomms8221 PMC4490369

[34] J. Liu , N. P. Wickramaratne , S. Z. Qiao , M. Jaroniec , Nat. Mater. 2015, 14, 763. 26201892 10.1038/nmat4317

[35] J. Tang , J. Liu , R. R. Salunkhe , T. Wang , Y. Yamauchi , Chem. Commun. 2016, 52, 505. 10.1039/c5cc07610b 26528620

[36] M. Zhou , C. Yang , K.‐Y. Chan , Adv. Energy Mater. 2014, 4, 1400840.

[37] D. Zhou , L. Yang , L. Yu , J. Kong , X. Yao , W. Liu , Z. Xu , X. Lu , Nanoscale 2015, 7, 1501. 25500995 10.1039/c4nr06366j

[38] M. Y. Song , D.‐S. Yang , K. P. Singh , J. Yuan , J.‐S. Yu , Appl. Catal. B 2016, 191, 202.

[39] S. Lee , D.‐H. Kwak , S.‐B. Han , E.‐T. Hwang , M.‐C. Kim , J.‐Y. Lee , Y.‐W. Lee , K.‐W. Park , Electrochim. Acta 2016, 191, 805.

[40] C. Liu , J. Wang , J. Li , R. Luo , X. Sun , J. Shen , W. Han , L. Wang , Carbon 2017, 114, 706.

[41] L. Lin , Q. Zhu , A. W. Xu , J. Am. Chem. Soc. 2014, 136, 11027. 25058390 10.1021/ja504696r

[42] K. Dietrich , E. Bonatz , R. Nastke , H. Herma , M. Walter , W. Teige , Acta Polym. 1990, 41, 91.

[43] J. Wei , Y. Liang , X. Zhang , G. P. Simon , D. Zhao , J. Zhang , S. Jiang , H. Wang , Nanoscale 2015, 7, 6247. 25779978 10.1039/c5nr00331h

[44] Y. Li , H. Tan , R. R. Salunkhe , J. Tang , L. K. Shrestha , B. P. Bastakoti , H. Rong , T. Takei , J. Henzie , Y. Yamauchi , K. Ariga , Chem. Commun. 2017, 53, 236. 10.1039/c6cc07360c 27921107

[45] X. Wang , C. Liang , S. Dai , Langmuir 2008, 24, 7500. 18547076 10.1021/la800529v

[46] C. Liang , S. Dai , J. Am. Chem. Soc. 2006, 128, 5316. 16620083 10.1021/ja060242k

[47] C. Liang , Z. Li , S. Dai , Angew. Chem. 2008, 120, 3754; 10.1002/anie.200702046 18350530

[48] K. Kailasam , Y.‐S. Jun , P. Katekomol , J. D. Epping , W. H. Hong , A. Thomas , Chem. Mater. 2010, 22, 428.

[49] T. Y. Ma , L. Liu , Z.‐Y. Yuan , Chem. Soc. Rev. 2013, 42, 3977. 23132523 10.1039/c2cs35301f

[50] F. Ma , H. Zhao , L. Sun , Q. Li , L. Huo , T. Xia , S. Gao , G. Pang , Z. Shi , S. Feng , J. Mater. Chem. 2012, 22, 13464.

[51] Y. Yao , B. Zhang , J. Shi , Q. Yang , ACS Appl. Mater. Interfaces 2015, 7, 7413. 25790324 10.1021/acsami.5b01233

[52] W. Huang , Y. Wang , G. Luo , F. Wei , Carbon 2003, 41, 2585.

[53] W. Niu , L. Li , X. Liu , N. Wang , J. Liu , W. Zhou , Z. Tang , S. Chen , J. Am. Chem. Soc. 2015, 137, 5555. 25860843 10.1021/jacs.5b02027

[54] S. Zhang , M. Zeng , J. Li , J. Li , J. Xu , X. Wang , J. Mater. Chem. A 2014, 2, 4391.

[55] S. Y. Hong , D. H. Chun , J.‐I. Yang , H. Jung , H.‐T. Lee , S. Hong , S. Jang , J. T. Lim , C. S. Kim , J. C. Park , Nanoscale 2015, 7, 16616. 26416550 10.1039/c5nr04546k

[56] Z. Yang , T. Zhao , X. Huang , X. Chu , T. Tang , Y. Ju , Q. Wang , Y. Hou , S. Gao , Chem. Sci. 2017, 8, 473. 28451194 10.1039/c6sc01819j PMC5298209

[57] H. Jiang , Y. Yao , Y. Zhu , Y. Liu , Y. Su , X. Yang , C. Li , ACS Appl. Mater. Interfaces 2015, 7, 21511. 26371772 10.1021/acsami.5b06708

[58] K. Ai , Y. Liu , C. Ruan , L. Lu , G. Lu , Adv. Mater. 2013, 25, 998. 23239109 10.1002/adma.201203923

[59] L. Zhang , J. Kim , E. Dy , S. Ban , K.‐c. Tsay , H. Kawai , Z. Shi , J. Zhang , Electrochim. Acta 2013, 108, 480.

[60] Y. Hu , J. O. Jensen , W. Zhang , S. Martin , R. Chenitz , C. Pan , W. Xing , N. J. Bjerrum , Q. Li , J. Mater. Chem. A 2015, 3, 1752.

[61] J. Xue , L. Zhao , Z. Dou , Y. Yang , Y. Guan , Z. Zhu , L. Cui , RSC Adv. 2016, 6, 110820.

[62] G. Ren , X. Lu , Y. Li , Y. Zhu , L. Dai , L. Jiang , ACS Appl. Mater. Interfaces 2016, 8, 4118. 26808226 10.1021/acsami.5b11786

[63] W. Chen , Z. Fan , X. Pan , X. Bao , J. Am. Chem. Soc. 2008, 130, 9414. 18576652 10.1021/ja8008192

[64] Y. Liu , H. Wang , D. Lin , J. Zhao , C. Liu , J. Xie , Y. Cui , Nano Res. 2017, 10, 1213.

[65] J. Yang , J. Hu , M. Weng , R. Tan , L. Tian , J. Yang , J. Amine , J. Zheng , H. Chen , F. Pan , ACS Appl. Mater. Interfaces 2017, 9, 4587. 28098443 10.1021/acsami.6b13166

[66] D. Deng , L. Yu , X. Chen , G. Wang , L. Jin , X. Pan , J. Deng , G. Sun , X. Bao , Angew. Chem. 2013, 125, 389; 10.1002/anie.201204958 23225769

[67] J. Wang , G. Wang , S. Miao , X. Jiang , J. Li , X. Bao , Carbon 2014, 75, 381.

[68] Z.‐Y. Wu , X.‐X. Xu , B.‐C. Hu , H.‐W. Liang , Y. Lin , L.‐F. Chen , S.‐H. Yu , Angew. Chem. 2015, 127, 8297;

