# Supplementary material for: Sub‐50 nm Iron–Nitrogen‐Doped Hollow Carbon Sphere‐Encapsulated Iron Carbide Nanoparticles as Efficient Oxygen Reduction Catalysts
Source: Adv Sci (Weinh). 2018 May 12;5(7):1800120. doi: 10.1002/advs.201800120 (PMC6051398; doi:10.1002/advs.201800120)
Supplement: Supplementary file 1 — Supplementary [file ADVS-5-1800120-s001.pdf]

Copyright WILEY-VCH Verlag GmbH & Co. KGaA, 69469 Weinheim, Germany, 2018.

ADVANCED SCIENCE
Open Access

Supporting Information

for *Adv. Sci.,* DOI: 10.1002/advs.201800120

Sub-50 nm Iron–Nitrogen-Doped Hollow Carbon Sphere-Encapsulated Iron Carbide Nanoparticles as Efficient Oxygen Reduction Catalysts

Haibo Tan, Yunqi Li, Jeonghun Kim, Toshiaki Takei, Zhongli Wang, Xingtao Xu, Jie Wang, Yoshio Bando, Yong-Mook Kang, Jing Tang,* and Yusuke Yamauchi**

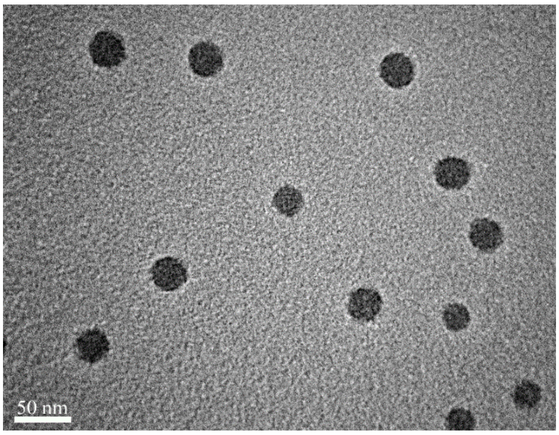

Figure S1. TEM image of triblock copolymer $PS_{(20,000)}$-*b*-$P2VP_{(15,000)}$-*b*-$PEO_{(27,000)}$ micelles. PS cores (dark spheres) are stained with 1.0 wt% phosphotungstic acid. The size of the PS core is *ca*. 31 nm.

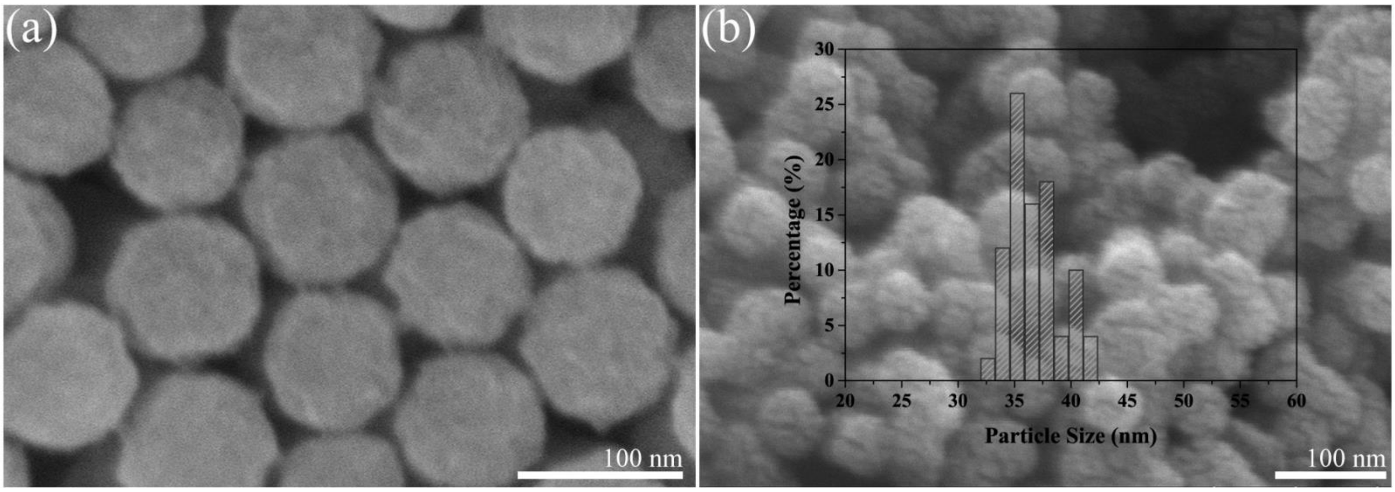

Figure S2. SEM images of (a) the as-prepared Fe_3C-Fe,N/C polymer precursor and (b) the corresponding carbonized product (Fe_3C-Fe,N/C-900) at 900 °C.

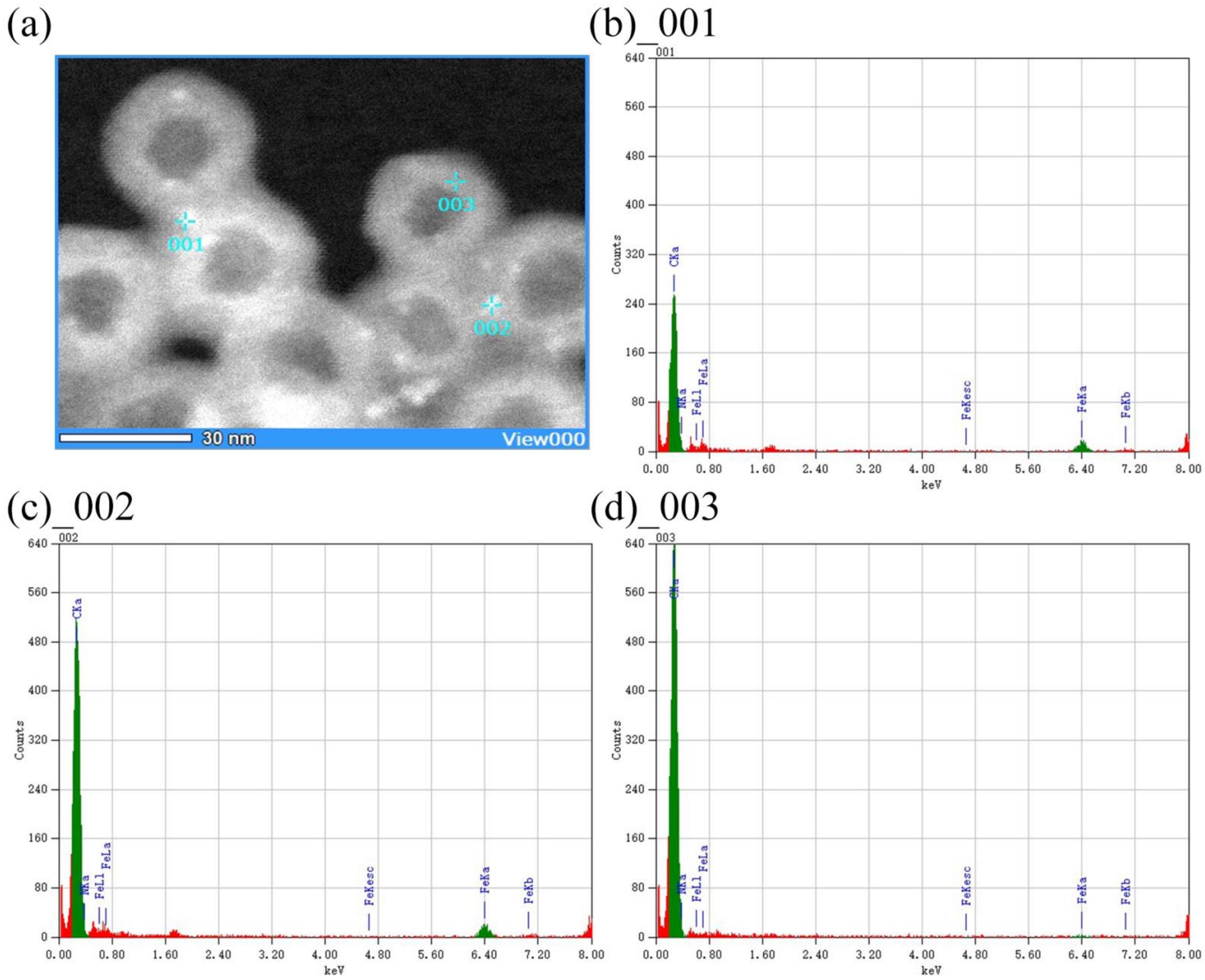

Figure S3. HAADF-STEM image of Fe_3C-Fe,N/C-900 hollow spheres and corresponding energy-dispersive X-ray (EDX) spectra of the selected spots.

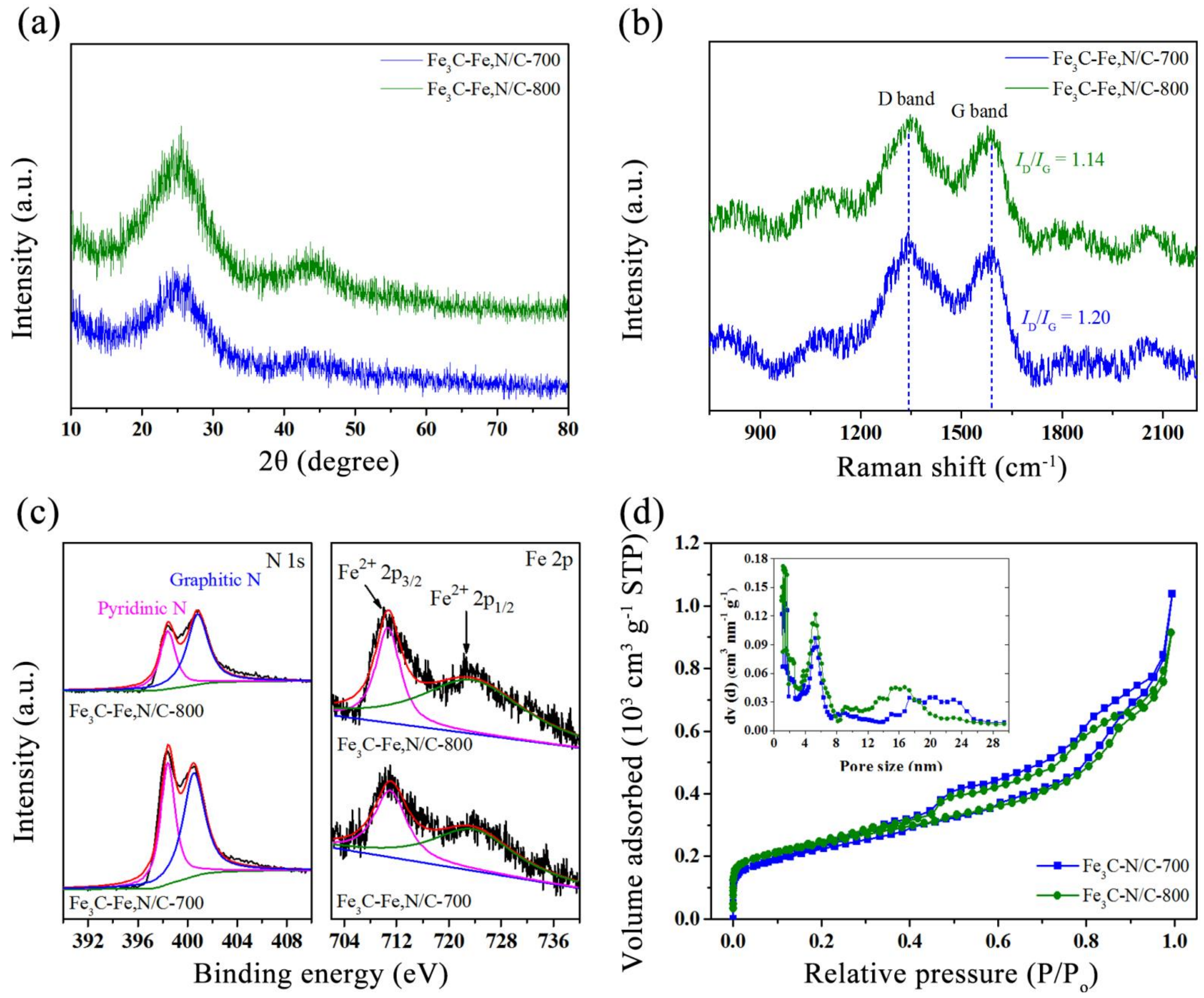

Figure S4. (a) XRD patterns, (b) Raman spectra, (c) high-resolution N 1s and Fe 2p XPS spectra, and (d) N_2 adsorption-desorption isotherms of Fe_3C-Fe,N/C-*x* (*x* = 700, 800).

Table S1. Physicochemical properties of the hollow Fe_3C-Fe,N/C-*x* and N/C-900 spheres

Samples	S_{BET} (m^2 g^{-1})	S_{Micro} (m^2 g^{-1})	S_{Micro}/ S_{BET}	V_{Pore} (cm^3 g^{-1})	V_{Micro} (cm^3 g^{-1})	V_{Micro}/V_{Pore}
N/C-900	1143.0	328.6	0.29	2.632	0.144	0.05
Fe_3C-Fe,N/C-700	805.1	71.6	0.09	1.306	0.024	0.02
Fe_3C-Fe,N/C-800	853.4	194.5	0.23	1.414	0.086	0.06
Fe_3C-Fe,N/C-900	879.5	259.6	0.30	1.590	0.143	0.09

S_{BET}: Specific surface area

S_{Micro}: Micropore surface area

V_{Pore}: Pore volume

V_{Micro}: Micropore volume

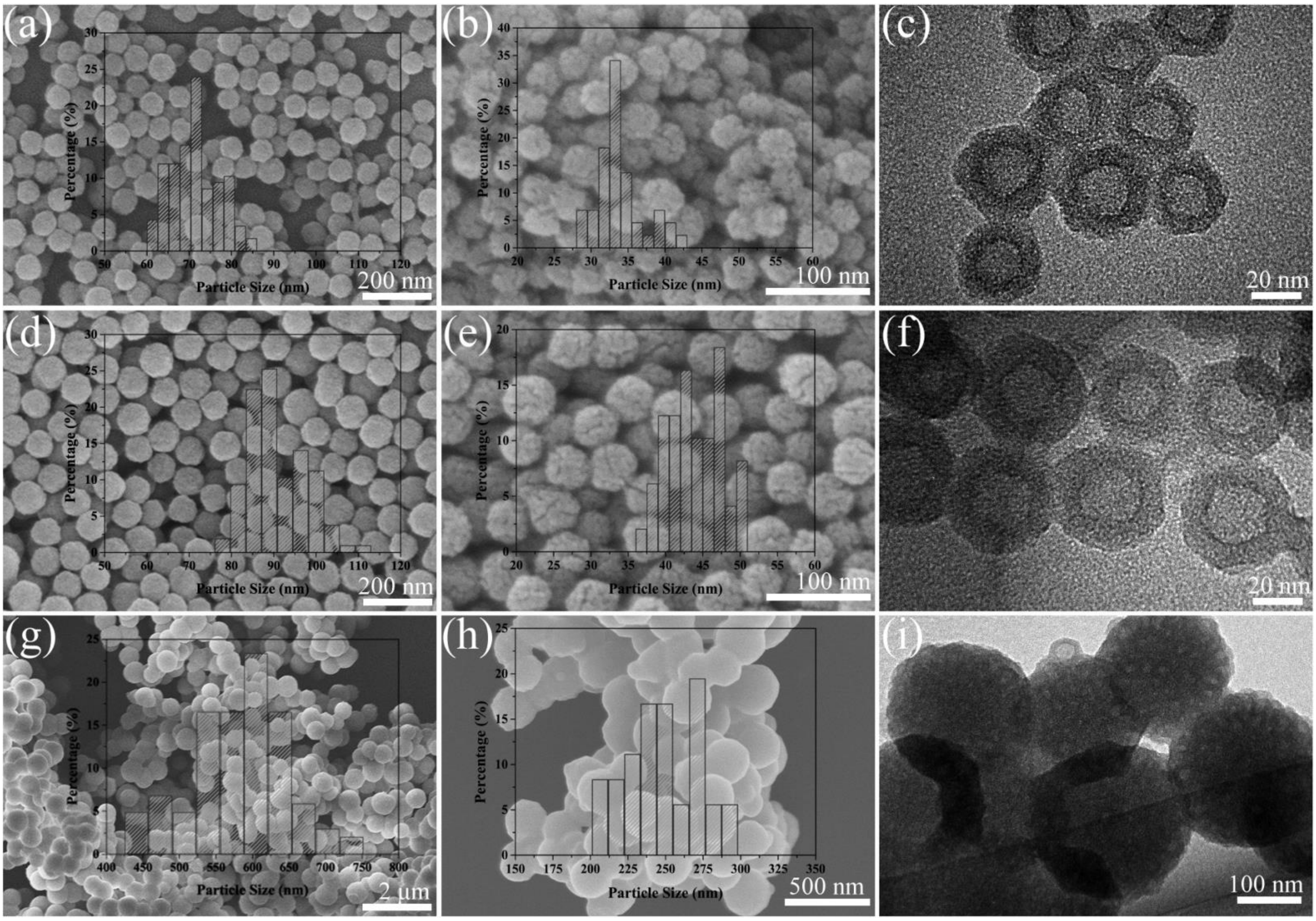

Figure S5. SEM and TEM images for the N/C precursor and carbonized products with different M-FR concentrations: (a) 0.03 M, (d) 0.05 M, and (g) 0.10 M, and their corresponding pyrolyzed products, (b, c), (e, f), and (h, i), at 800 °C.

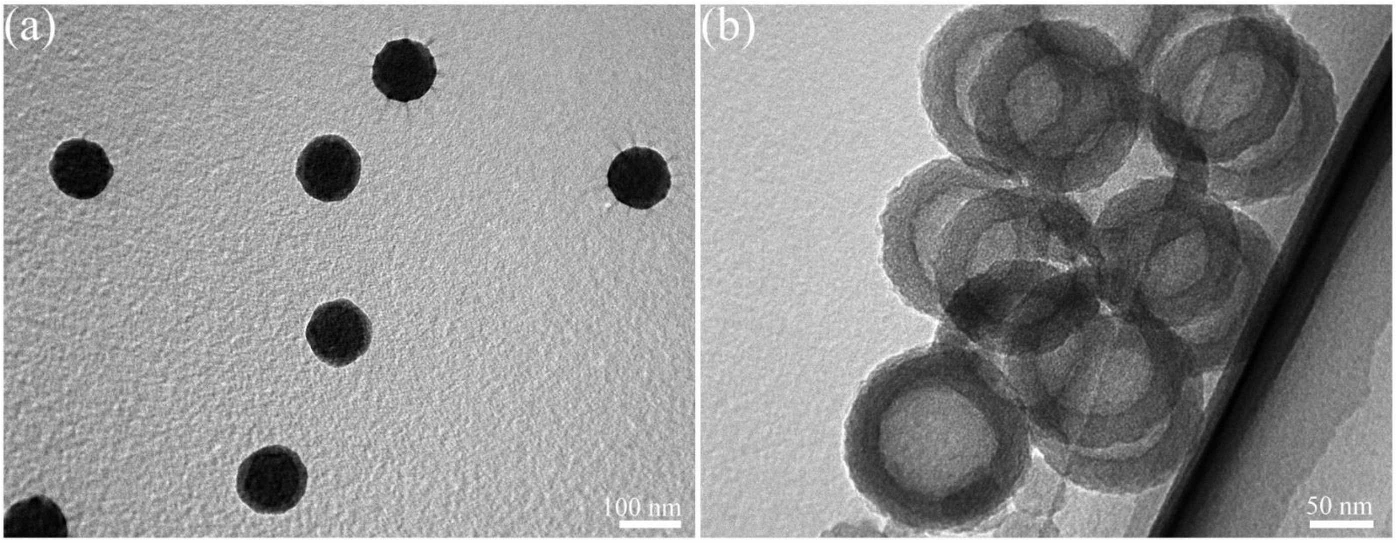

Figure S6. TEM images of micelles of triblock copolymer $PS_{(45,000)}$-*b*-$P2VP_{(26,000)}$-*b*-$PEO_{(82,000)}$ and hollow N/C pyrolyzed at 800 °C. The PS cores (dark spheres) were stained with 1.0 wt% phosphotungstic acid.

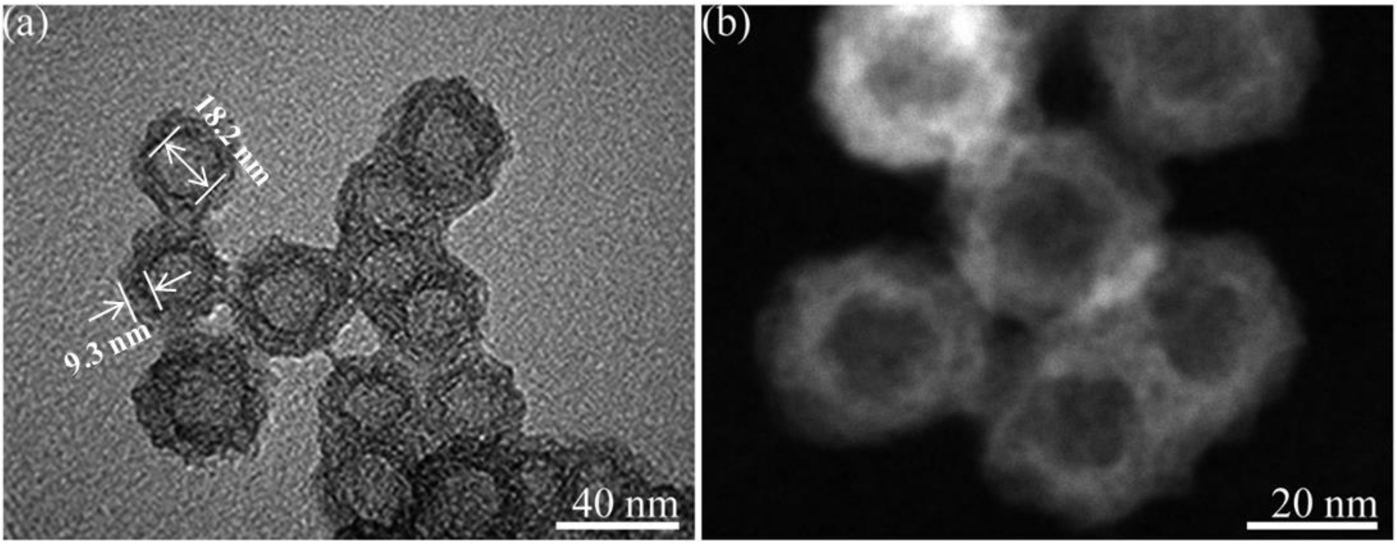

Figure S7. (a) TEM and (b) HAADF-STEM images of N/C-900 hollow spheres.

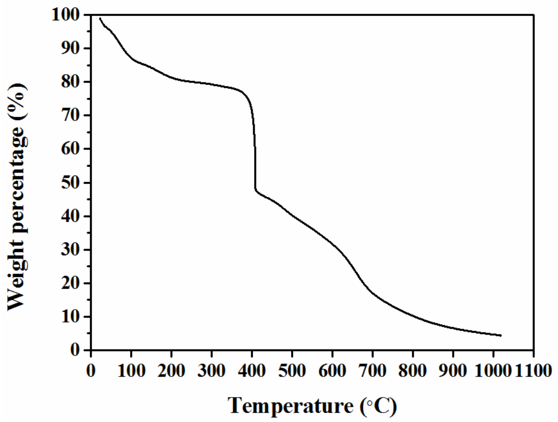

Figure S8. TGA curve of M-FR-coated triblock polymer micelles at a ramping rate of 5 °C min^{-1} under a N_2 atmosphere.

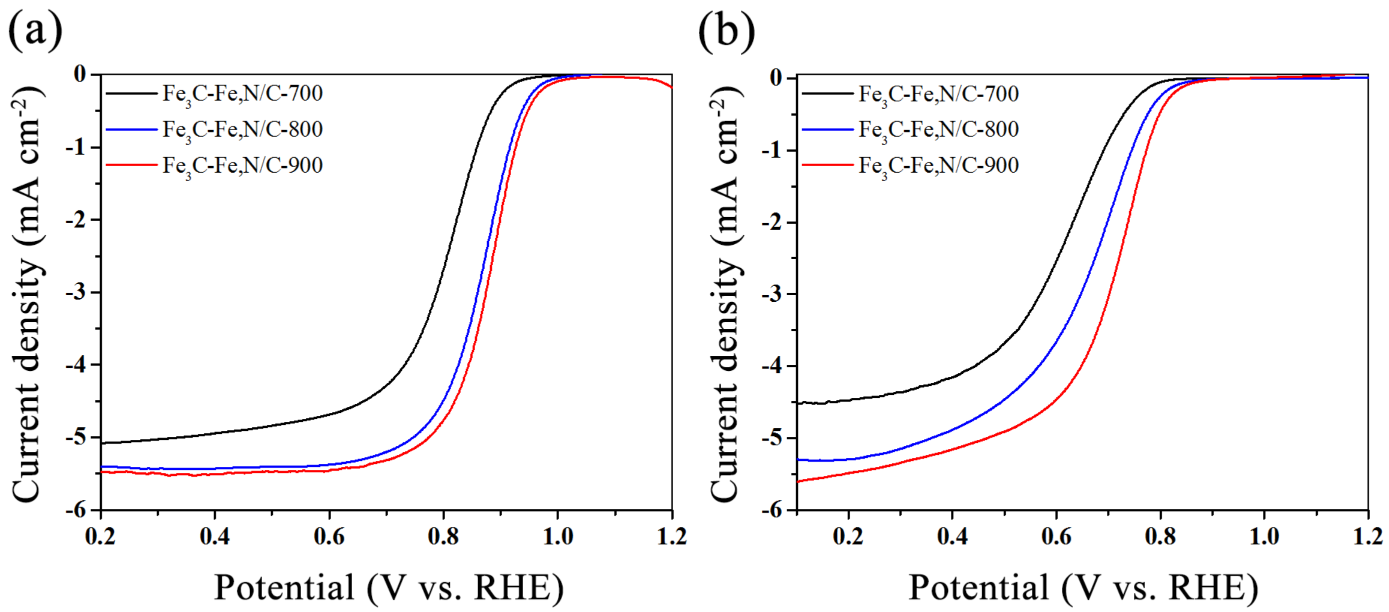

Figure S9. Linear sweep voltammetry (LSV) curves of Fe_3C-Fe,N/C-*x* (*x* = 700, 800, 900) at a scan rate of 10 mV s^{-1} under a rotation speed of 1600 rpm in O_2/N_2-saturated (a) 0.1 M KOH and (b) 0.1 M $HClO_4$.

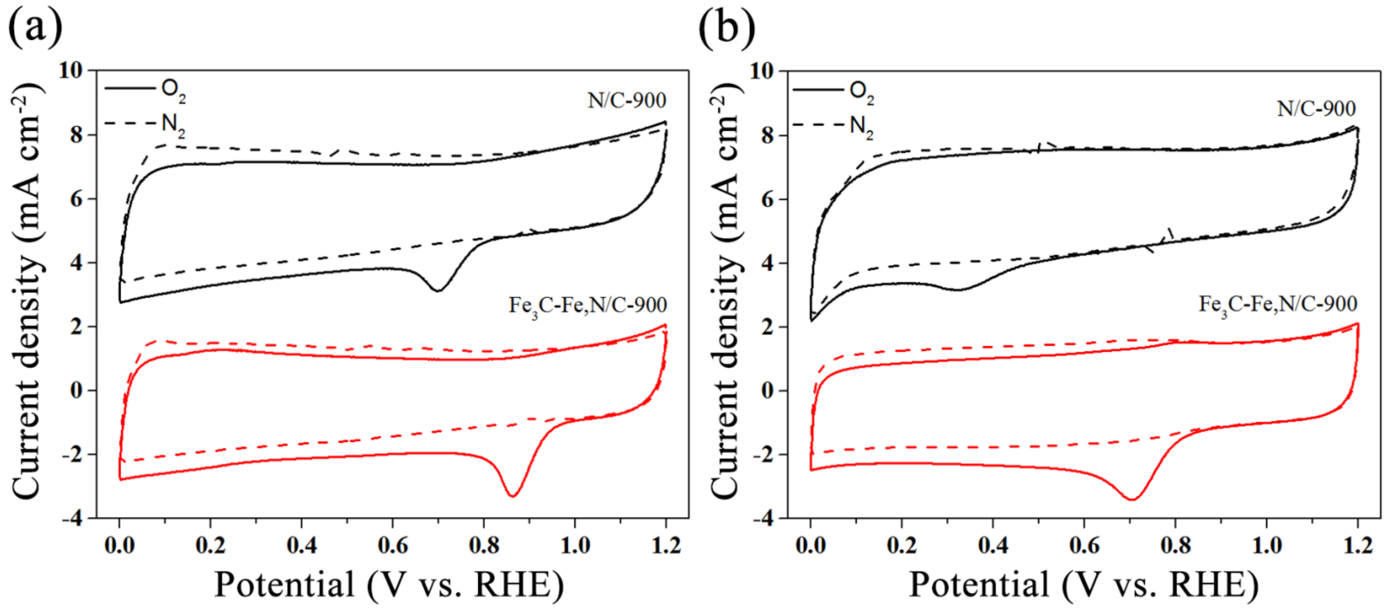

Figure S10. Cyclic voltammograms of Fe_3C-Fe,N/C-900 and N/C-900 in O_2/N_2-saturated (a) 0.1 M KOH and (b) 0.1 M $HClO_4$, respectively, at a scan rate of 50 mV s^{-1}. For clarity, the current density for N/C-900 is offset vertically by 6 mA cm^{-2}.

Table S2. Summary of ORR activities of Fe_3C-Fe,N/C-900 and recently reported Fe_3C-based catalysts in 0.1 M KOH (electrode rotation speed of 1600 rpm)

**Catalysts**	**Catalyst loading ($mg\ cm^{-2}$)**	**LSV scan rate ($mV\ s^{-1}$)**	**Half-wave potential (V *vs.* RHE)**	**Current density (mA cm^{-2}) (potential *vs.* RHE)**	**Electron transfer number (potential *vs.* RHE)**	**Reference**
Fe_3C-Fe,N/C-900	0.20	10	0.881	5.53 (at 0.45 V)	3.98-4.05 (0.4 to 0.7 V)	This work
Fe_3C/C-800	0.60	10 (900 rpm)	0.83	*ca.* 3.9 (at 0.45 V)	3.8-4.0 (0.3 to 0.6 V)	[1]
Fe/Fe_3C @C/RGO	0.53	5	0.93	*ca.* 9.6 (at 0.45 V)	3.52-3.08 (0.5 to 0.7 V)	[2]
Fe_3C@NG 800-0.2	0.20	10	0.81	*ca.* 5.1 (at 0.45 V)	3.94 (0 to 0.8 V)	[3]
Fe-N-CNFs	0.60	10	-0.14 [*vs.* Ag/AgCl/KCl (3.5 M)]	*ca.* 5.14 [at -0.6 V *vs.* Ag/AgCl/KCl (3.5 M)]	3.93-3.95 [-0.4 to -0.6 V *vs.* Ag/AgCl/KCl (3.5 M)]	[4]
Fe_3C/NG -800	0.40	5	0.86	*ca.* 5.68 (at 0.45)	3.89-4.0 (0 to 0.8 V)	[5]
Fe/Fe_3C @N-rGO	0.25	5	0.82	*ca.* 5.35 (at 0.45 V)	*ca.* 4.0 (0.4 to 0.7 V)	[6]
F-PNG	0.10	5	-0.187 (*vs.* Ag/AgCl/KCl)	*ca.* 4.33 (at -0.6 V *vs.* Ag/AgCl/KCl)	*ca.* 3.98 (-0.3 to -0.7 V *vs.* Ag/AgCl/KCl)	[7]
Fe–C/ NG-10% -700-AL	0.28	10	0.793	6.15 (at 0.45 V)	Unknown	[8]
Fe_3C@NC NF-900	0.41	10	-0.121 (*vs.* SCE)	4.51 (Unknown)	3.8 (at -0.5 V *vs.* SCE)	[9]
Fe_3C@C -900	0.30	10	0.80	*ca.* 5.18 (at 0.45 V)	3.98-4.05 (0 to 0.5 V)	[10]

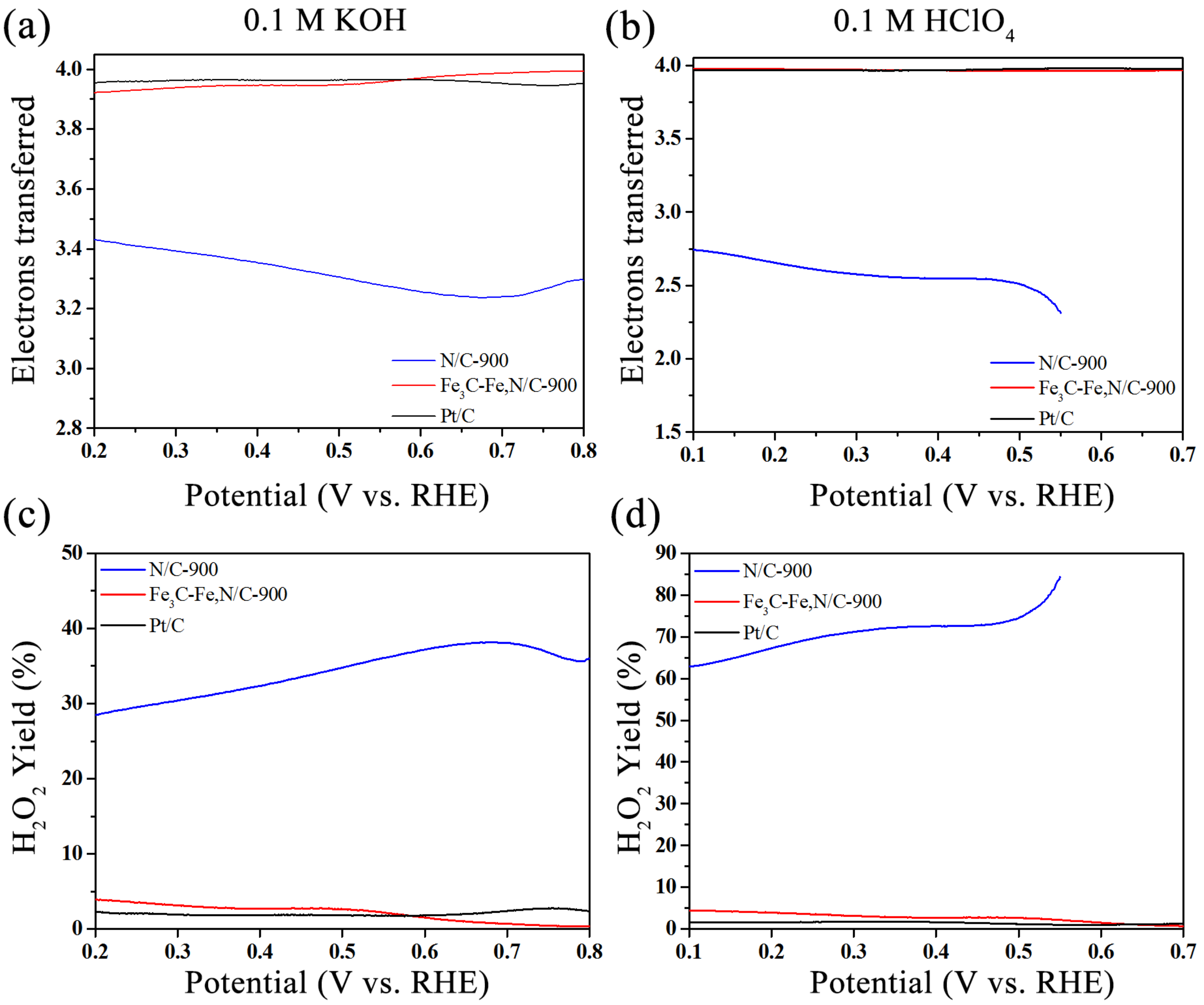

Figure S11. (a, b) Electron transfer number and (c, d) peroxide yield (H_2O_2) calculated based on disk-ring currents of N/C-900, Fe_3C-Fe,N/C-900, and Pt/C in O_2-saturated 0.1 M KOH and 0.1 M $HClO_4$ at a scan rate of 10 mV s^{-1} under a rotation speed of 1600 rpm.

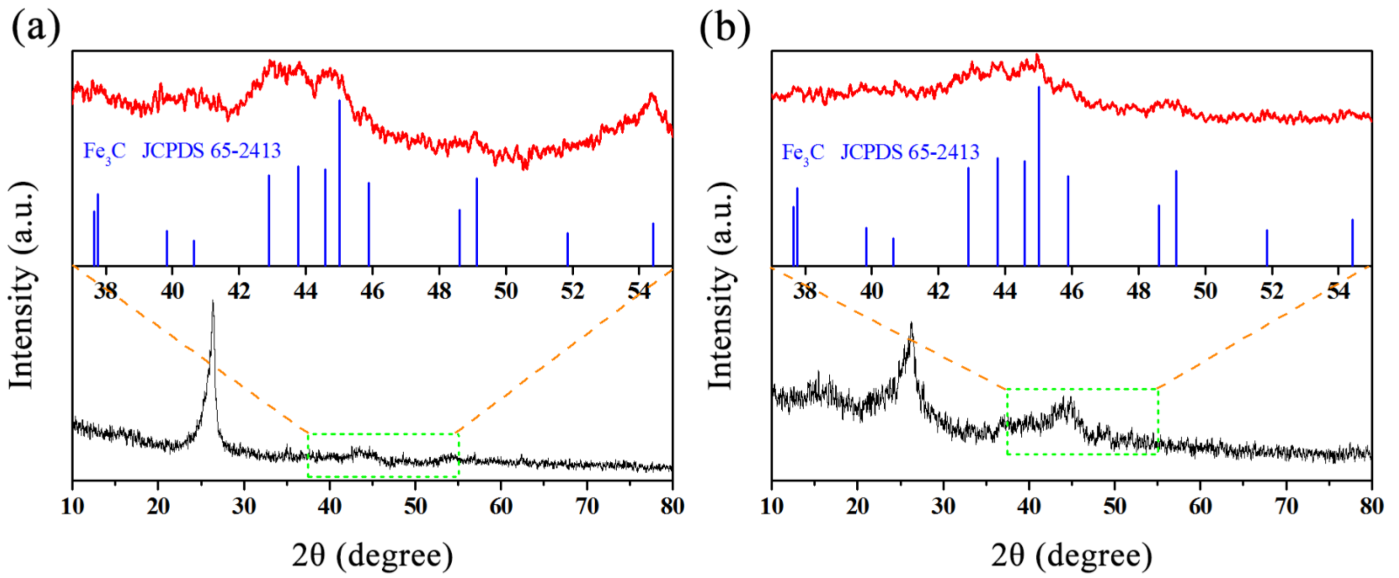

Figure S12. XRD patterns of Fe_3C-Fe,N/C-900 after 5000 cycles in O_2/N_2-saturated (a) 0.1 M KOH and (b) 0.1 M $HClO_4$. Only 25 μg of Fe_3C-Fe,N/C-900 powder was deposited on the surface of a GC electrode for the general cycling measurement, which is insufficient for the XRD measurement. To obtain more powder, 2.5 mg of Fe_3C-Fe,N/C-900 powder was deposited on a 1×1 cm carbon paper (Toray, TGP-H-090) and removed from the carbon paper after the cycling measurement by sonication. After cycling for the XRD measurement, the Fe_3C-Fe,N/C-900 powder was mixed with graphite; the sharp diffraction peaks at 26° are mainly attributed to graphite.

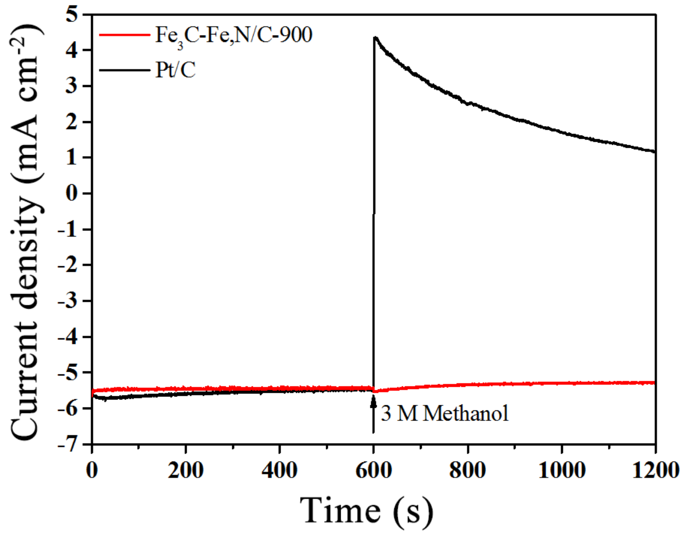

Figure S13. Chronoamperometric response with 3 M methanol of Fe_3C-Fe,N/C-900 and Pt/C at 0.65 V and 1600 rpm in O_2-saturated 0.1 M KOH.

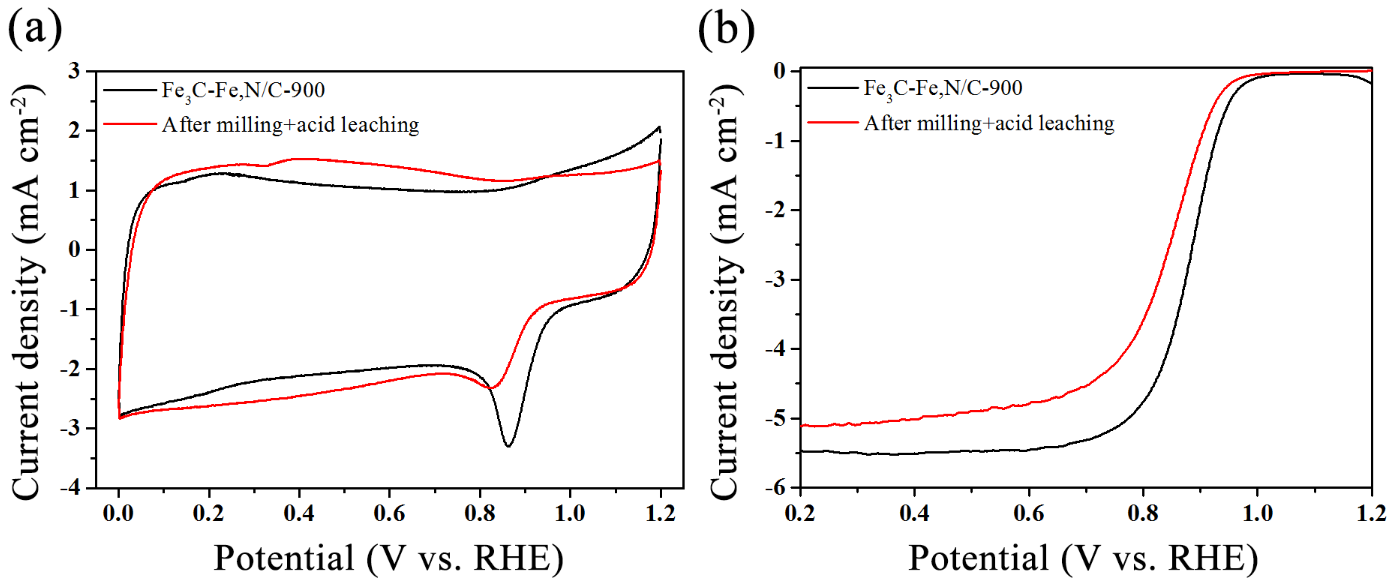

Figure S14. (a) CV and (b) LSV curves of Fe_3C-Fe,N/C-900 and milled Fe_3C-Fe,N/C-900 after hot acid leaching in O_2-saturated 0.1 M KOH.

References

[1] Y. Hu, J. O. Jensen, W. Zhang, L. N. Cleemann, W. Xing, N. J. Bjerrum, Q. Li, *Angew. Chem.* **2014**, *126*, 3749; *Angew. Chem. Int. Ed.* **2014**, *53*, 3675.

[2] Y. Hou, T. Huang, Z. Wen, S. Mao, S. Cui, J. Chen, *Adv. Energy Mater.* **2014**, *4*, 1400337.

[3] H. Jiang, Y. Yao, Y. Zhu, Y. Liu, Y. Su, X. Yang, C. Li, *ACS Appl. Mater. Interfaces* **2015**, *7*, 21511.

[4] Z.Y. Wu, X.X. Xu, B.C. Hu, H.W. Liang, Y. Lin, L.F. Chen, S.H. Yu, *Angew. Chem.* **2015**, *127*, 8297; *Angew. Chem. Int. Ed.* **2015**, *54*, 8179.

[5] M. Xiao, J. Zhu, L. Feng, C. Liu, W. Xing, *Adv. Mater.* **2015**, *27*, 2521.

[6] Y. Liu, H. Wang, D. Lin, J. Zhao, C. Liu, J. Xie, Y. Cui, *Nano Res.* **2017**, *10*, 1213.

[7] Y. Lai, W. Chen, Z. Zhang, Y. Qu, Y. Gan, J. Li, *Electrochim. Acta* **2016**, *191*, 733.

[8] J. Xue, L. Zhao, Z. Dou, Y. Yang, Y. Guan, Z. Zhu, L. Cui, *RSC Adv.* **2016**, *6*, 110820.

[9] G. Ren, X. Lu, Y. Li, Y. Zhu, L. Dai, L. Jiang, *ACS Appl. Mater. Interfaces* **2016**, *8*, 4118.

[10] A. Kong, Y. Zhang, Z. Chen, A. Chen, C. Li, H. Wang, Y. Shan, *Carbon* **2017**, *116*, 606.
